# Importance of mitigation measures for hospital transmission of SARS-CoV-2 at the onset of the epidemic: the experience of Brescia, Northern Italy

**DOI:** 10.1007/s15010-021-01692-9

**Published:** 2021-09-15

**Authors:** Valentina Marchese, Beatrice Formenti, Gloria Cola, Natalia Gregori, Elisa Albini, Giuseppe De Palma, Irene Possenti, Marco Scala, Francesco Castelli, Alberto Matteelli

**Affiliations:** 1 University Department of Infectious and Tropical Diseases, WHO Collaborating Centre for TB/HIV Collaborative Activities and for TB Elimination Strategy, University of Brescia, ASST Spedali Civili of Brescia, Brescia, Italy; 2 Department of Infectious and Tropical Diseases, University of Brescia, Piazza Spedali Civili, 1, 25125 Brescia, Italy; 3 Section of Occupational Health, Department of Experimental and Applied Medicine, University of Brescia, Brescia, Italy; 4 Section of Public Health and Human Sciences, Department of Medical and Surgical Specialties, Radiological Sciences, and Public Health, University of Brescia, Brescia, Italy; 5 Department of Statistical Sciences “Paolo Fortunati”, University of Bologna, Bologna, Italy; 6 Department of Environmental Health Sciences, Instituto di Ricerche Farmacologiche Mario Negri IRCCS, Milan, Italy

**Keywords:** COVID-19, SARS-CoV-2, Health-care workers, Hospital infection, Mitigation measures, Nosocomial infection

## Abstract

**Purpose:**

Since the first Italian case of SARS-CoV-2 was detected in Lombardy (Northern Italy)  Italy quickly became one of the worst-affected European countries, with a severe impact on health-care workers (HCWs). In the first epidemic, HCWs accounted for 12% of all national COVID-19 cases. We evaluated the burden of COVID-19 among HCWs and other non-health-care workers (nHCWs) in a large Italian hospital.

**Methods:**

From March 1st to May 31st 2020, we performed a retrospective study at ASST Civil Hospital, in the Province of Brescia, Lombardy. The study population included all hospital personnel (*n* = 9265), categorized by professional status.

**Results:**

A SARS-CoV-2 test was performed in 3572 workers (38.5%), with a positive result in 552 (5.9% of all hospital personnel). The temporal trend of SARS-CoV-2 cases in hospital staff broadly reflected that in the community, with a great majority of infections occurred during March 2020 (87.7%). From April onward, a steep decrease of positive cases was observed among hospital personnel, while in the community the decrease was much slower. Medical doctors (8.9%) and nurses (8.5%) were the most affected professional categories with a significantly higher risk of SARS-CoV-2 infection (OR 1.436 and OR 1.410, respectively *p* < 0.0001). HCWs in COVID-19 units presented a significantly higher risk of infection compared to HCWs in non-COVID units (*p* < 0.001).

**Conclusion:**

HCWs were severely affected by the COVID-19 epidemic, probably associated with an overwhelming burden of work and lack of preparedness in prevention of nosocomial transmission of the infection. The rapid decrease of COVID-19 spread in the hospital, registered before the one in the community, suggests that the adopted preventive measures were effective.

## Background

On 11 March 2020, CoronaVirus Disease 2019 (COVID-19), a new respiratory infectious disease caused by severe acute respiratory syndrome coronavirus 2 (SARS-CoV-2), was declared by WHO as a pandemic [1]. With over 113 million confirmed cases and 2.5 million deaths worldwide as of 1st of March 2021 [2], COVID-19 has posed enormous challenges to health systems globally. The sudden onset of the pandemic found hospitals and health-care workers (HCWs) unprepared and determined the need to convert significant resources in response to the epidemic. HCWs and all hospital personnel faced an increased risk of SARS-CoV-2 infection due to their professional exposure. Shortages of personal protective equipment (PPE) and a delay in the application of strict isolation measures [3–6] were common at this stage. According to WHO data [7], in September 2020 HCWs accounted for 14% of worldwide COVID-19 cases, reaching 35% in some countries, while representing less than 3% of the general population. In the first report of 138 COVID-19-positive patients from Wuhan, China, 29% of the cases were HCWs [8], while in a cohort study from a single hospital in Spain, 11% of HCWs had COVID-19 during the first months of the epidemic [9].

Italy registered its first case of SARS-CoV-2 on February 21, 2020, and soon became one of the worst-affected European countries. HCWs accounted for 12% of all national cases by June 2020 [10].

HCWs represent one of the most precious resources in the fight against the pandemic; focusing research on prevention and control strategies for SARS-CoV-2 nosocomial infection is essential for many reasons. First, infected HCWs and hospital personnel could transmit the virus to vulnerable patients and to close contacts both in hospital and in the community, expanding COVID-19 spread [6]. Moreover, high rates of infection among health personnel inevitably lead to staff shortage weakening the health-care system [11]. This provides the rationale for prioritizing protective interventions among HCWs [5, 6, 11].

We studied the impact of SARS-CoV-2 infection among hospital personnel in a tertiary hospital of Brescia, Lombardy (6.3% of all Italian cases, as on May 31st, 2020) to identify factors associated with a higher risk of SARS-CoV-2 nosocomial transmission.

## Methods

### Population and study period

We performed a retrospective analysis of data collected between March 1st and May 31st, 2020, at the Local Health and Social Organization (ASST) Civil Hospital of Brescia, a public tertiary referral University hospital. The hospital provides care to the entire municipality at three different sites: Civile Hospital that is the main city hospital, and the decentralized Montichiari Hospital, and Gardone Val Trompia Hospital. Our study population included all hospital personnel working during the study period, categorized by professional status: non-health-care workers (nHCWs), including personnel without any contact with patients [i.e., pharmacists, engineers, sterilization personnel, drivers, priests, secretaries, food service staff, administrative staff (manager, accountant, human resources office, legal affairs)]; and HCWs, including medical doctors (MDs), residents, nurses and midwives, health-care assistants (HCAs) and technicians (i.e., physiotherapists and X-ray technicians). Students were excluded from the analysis. On May 31st, 2020, there were 9265 workers employed by the ASST Civil Hospital of Brescia, of whom 2497 (26.9%) were nHCWs and 6,345,768 (68.5%) were HCWs, including 2481 nurses (26.8%) and 1209 HCAs.

### Diagnostic method and definition of COVID-19

According to the hospital protocol [12], during the study period any nHCW and HCW was tested for SARS-CoV-2 if presenting with: body temperature > 37.5 °C, fatigue, headache, myalgia and at least one among cough, sore throat and dyspnea, or history of close contact with a confirmed case of COVID-19 (defined by WHO’s guidelines [13]). Testing was based on nose-pharyngeal (NP) swabs for detection of SARS-CoV-2 nucleic acid by specific real-time polymerase chain reaction (RT-PCR). A confirmed case of COVID-19 was defined by a swab positive for SARS-CoV-2 RT-PCR. Every SARS-CoV-2 positive result from any accredited laboratory had to be notified to the occupational health services of the hospital.

### Variables and data collection

We retrospectively collected data regarding confirmed COVID-19 cases among hospital employees mining the databases of the Occupational Health Department and Human Resources Department. For each infected case we extracted demographic data (age and sex), professional status and site of work. The database was anonymized.

Hospital employees were categorized according to their professional status and place of work during the study period. Units of the main Hospital Civile were divided into non-COVID units and COVID dedicated units (*n* = 16).

### Questionnaire-based survey

To get an insight into the characteristics of COVID-19 spread within the hospital, we performed a voluntary online questionnaire sub-study targeting COVID-19-affected employees. The questionnaire was available from the beginning of June to 31 July 2020. The survey covered the following areas: (i) symptoms and time of onset; (ii) suspected intra- or extra-hospital source of contagion: patients, colleagues, family members, others; (iii) circumstances and location of presumptive contact with the source of contagion: invasive and non-invasive procedures generating droplets, indoor place, use and type of mask, and kind of close contact [14]*.*

### Statistical analysis

Descriptive analysis of the data was performed by determining the incidence rate of SARS-CoV-2 infection in HCWs stratified by professional status and operational unit (OU). Categorical variables were summarized as numbers and percentages and continuous variables were expressed as mean and standard deviation (SD). The outcome was correlated with different categorical variables using the Chi-squared test, with *p* < 0.05 taken to indicate significance. Possible associations between variables and the primary outcome were explored by odds ratios (OR). ORs were presented with 95% CIs, calculated using the normal approximation (Wald). All analyses were performed using SAS version 9.4 (SAS Institute, Cary, NC).

## Results

### Incidence of COVID-19 by professional role of hospital employees

During the study period, 3572 workers (38.5%) were tested for SARS-CoV-2, of whom 552 had confirmed infection, accounting for 5.9% of all employees. The mean age of those infected was 46.23 (SD ± 11.1), and 415 (70%) were female. The categories receiving the higher number of tests were nurses (*n* = 1424; 57.3%), along with HCAs (*n* = 687; 56.8%) and MDs (*n* = 594; 50.8%). The highest positivity rates were observed among MDs (8.9%, 104 positive cases) and nurses (8.5%, 213 positive cases), followed by technicians (7.6%, 47 positive cases) and HCAs (6.9%, 84 positive cases). As expected, nHCWs presented the lower proportion of PCR tests performed (*n* = 242, 9.7%), with the lowest incidence rate (< 2%). Applying a logistic regression model to our data, all the HCWs except for the residents showed a significantly higher risk of being infected by SARS-CoV-2 than nHCWs (*p* < 0.05) (Table 1).

**Table 1:** Proportion of hospital personnel tested for SARS-CoV-2, positivity rate, and proportion positive over the population, according to professional status

Professional status	Total, *N* (%)	Tested, *N* (%)	Positive rate, *N* (%)	Incidence rate, %	*p* value	OR (CI)
nHCWs	2497 (26.9)	242 (9.7)	46 (19)	1.8	“Ref”	“Ref”
MD	1167 (12.6)	594 (50.9)	104 (17.5)	8.9	< 0.0001*	5,430 (3.779–7.802)
Residents	872 (9.4)	323 (37)	53 (16.4)	6.0	0.88	3.693 (2.451–5.565)
Nurses	2481 (26.8)	1424 (57.4)	213 (14.9)	8.5	< 0.0001*	5.332 (3.823–7.437)
HCAs	1209 (13)	687 (56.8)	84 (12.2)	6.9	0.04*	4.490 (3.092–6.520)
Technicians	616 (6.6)	265 (43)	47 (17.7)	7.6	0.05*	4.714 (3.086–7.2)
Unknown	423 (4.6)	40 (9.4)	5 (12.5)	1.2		
Total	9265 (100)	3575 (38.6)	552 (15.4)	5.9		

*OR* odds ratio, *CI* confidence interval

*p* value from *χ*^2^ test

HCWs of Infectious Disease Department, the first department accepting confirmed COVID-19 patients, had a positive-NP swab rate of 25% with a significantly increased risk of COVID-19 infection compared to other departments (OR 1.864; CI 1.164–2.986; *p* value 0.0009).

In a second step of the analysis, we gathered HCAs, residents, technicians and workers of unknown status in a unique category called “other status” and we compared it with nHCWs, nurses and doctors. This logistic regression model confirmed a significantly lower proportion of SARS-CoV-2 infection for nHCWs (OR 0.264; CI 0.189–0.370; *p* value < 0.0001) and an increased proportion of infection among medical doctors (OR 1.436; CI 1.120–1.841; *p* value < 0.0001) and nurses (OR 1.410; CI 1.151–1.727; *p* value < 0.0001) (Table 2).

**Table 2:** Logistic regression analysis of the probability of a positive test and incidence rate of SARS-CoV-2 in hospital personnel according to occupational exposure

Professional status	Total, *N* (%)	Tested, *N* (%)	Positive rate, *N* (% over tested)	Incidence rate, %	*p* value	OR (CI)
Other status	3120 (33.7)	1315 (42.1)	189 (1 4.3)	6.0	“Ref”	“Ref”
MD	1167 (12.6)	594 (50.9)	104 (17.5)	8.9	< 0.0001	1.436 (1.120–1.841)
Nurses	2481 (26.8)	1424 (57.4)	213 (14.9)	8.5	< 0.0001	1.410 (1.151–1.727)
nHCWs	2497 (26.9)	242 (9.7)	46 (19)	1.8	< 0.0001	0.264 (0.189–0.370)

*OR* odds ratio, *CI* confidence interval

*p* value from *χ*^2^ test

### Temporal trend of COVID-19 infections

Among hospital employees, the first confirmed case of COVID-19 was diagnosed on March 2. Of all positive-NP swabs detected during the study period (*n* = 552), the great majority (484, 87.7%) occurred in March, followed by a sharp decrease in April (59, 10.7%) and May (9, 1.6%). Monthly incidence rates were 5.2%, 0.63% and 0.1%.

**Fig. 1:**
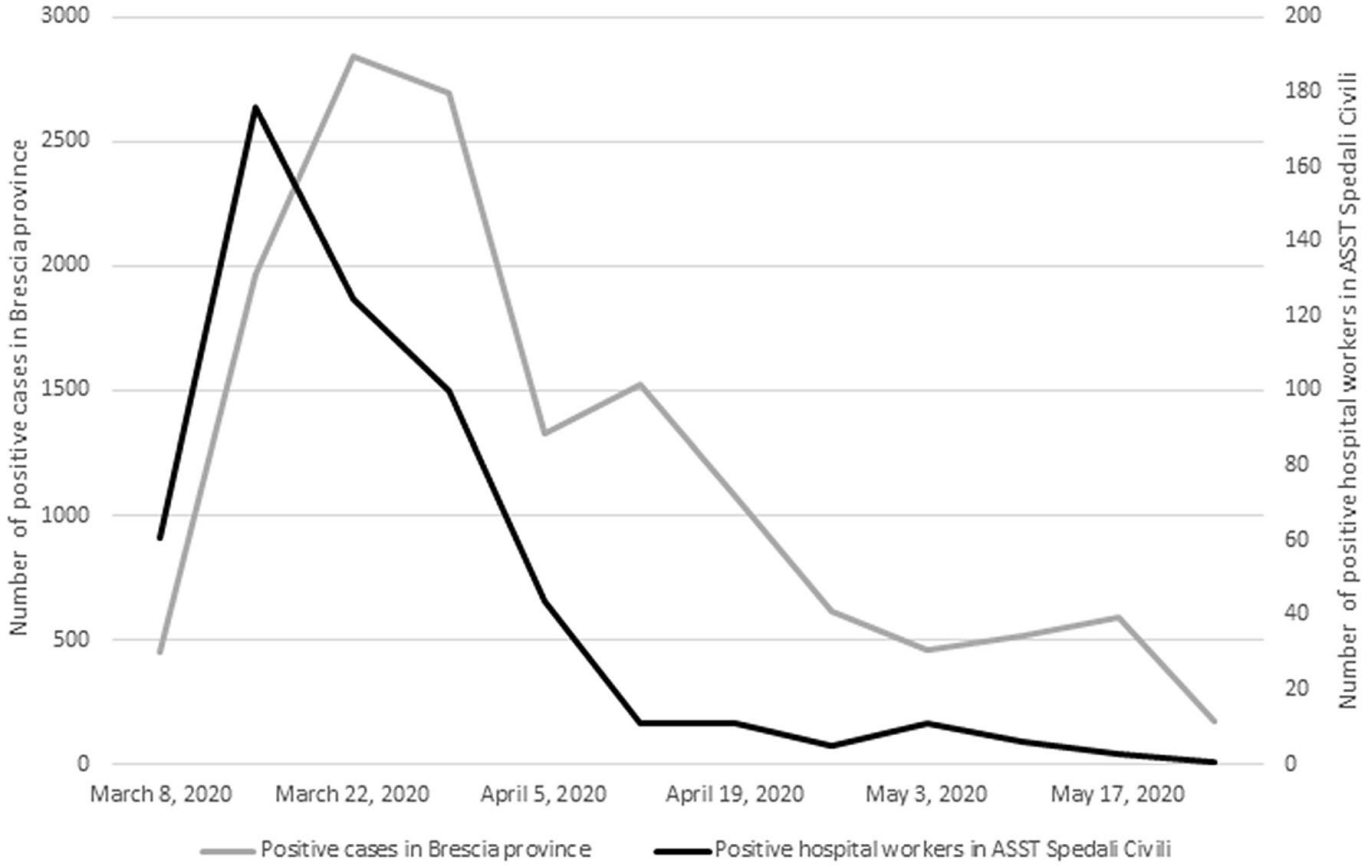
Weekly cases of SARS-CoV-2 infection among hospital personnel of ASST Spedali Civili and Brescia community

Figure 1 shows the number of confirmed cases among hospital workers per week, along with the total number of cases notified in Brescia during the study period. The peak of the infection was reached earlier, and flattened earlier, among hospital personnel than in the general population. Among the latter, incidence continued to be high during April and May 2020.

### Incidence of SARS-CoV-2 infection among hospital personnel based on departments of exposure

The analysis of the cumulative incidence of SARS-CoV-2 infection by departments of exposure was performed only for the main hospital personnel (8121 workers, more than 80% of the ASST staff). Most workers were employed in non-COVID units (6618 staff, 81.4% vs. 1503, 18.5%) that recorded a cumulative incidence rate of 4.4%, as opposed to COVID units where the incidence was 11%.

Staff of COVID units had a significantly higher risk of infection compared to staff of non-COVID units (OR 2.74, CI 2.25–3.34, *p* value < 0.001).

### Perceived routes of COVID-19 transmission through online voluntary questionnaire

i. *Symptoms—*Of the 552 infected workers, 343 (62.1%) completed the questionnaire section regarding symptoms before the diagnosis of COVID-19. The vast majority (72.0%) of the respondents declared to have had symptoms before or at the moment of the test, such as anosmia, cough, myalgia and fatigue. Overall, 66.8% had a body temperature above 37 °C. Twenty-eight percent of the respondents declared not to have had any symptoms before COVID-19 diagnosis and had been tested because of a contact with a patient with COVID-19.
ii. *Presumptive source of transmission—*Out of 552 positive workers, 279 (50.5%) answered regarding the presumptive source of transmission and its modalities. The great majority (88.1%) assumed to have been infected by SARS-CoV-2 in the hospital setting. Among them, almost 40% reported a colleague as a possible source of infection, who later was diagnosed with COVID-19; on the other hand, 31.9% of respondents perceived to have acquired the infection from hospitalized patients, although most of them (76.4%) did not report performing invasive investigations (i.e., intubation and aspiration). Nearly 17% of respondents did not identify the source, since they have had contacts with both infected patients and colleagues (Table 3).
iii. *Use of mask at the time of infection—*Two hundred and thirteen (38.6%) of 552 infected employees answered regarding the use of masks at the time of infection. Almost 30% reported the use of FFP2/3 masks, while surgical masks were reportedly worn by 38.0% of the respondents. The remainder stated not to have worn any type of mask at the time of the presumptive contagion. More than 75% of the positive workers who had unprotected contact had also got in touch with colleagues (Table 4).

**Table 3:** Presumptive source of transmission

Presumptive source of contagion	Positive interviewees, *N* (%)
Colleague	110 (39.4)
Patient	89 (31.9)
Non-invasive procedure	68 (76.4)
Invasive procedure	21 (23.6)
Patient and colleague	47 (16.9)
Out of hospital	33 (11.8)
Total	279

**Table 4:** Use of PPE among infected interviewees

Mask	Positive interviewees, *N* (%)
FFP2/3 mask	61 (28.6)
Surgical mask	81 (38.0)
No mask	71 (33.4)
Contact with patient	12 (16.9)
Contact with colleague	54 (76.0)
Unknown	5 (7.1)
Total	213

## Discussion

Brescia was one of the first areas to be devastated by the SARS-CoV-2 epidemic in Europe, with a cumulative incidence of > 500/100,000 as on June 3rd, 2020 [10]. The temporal trend of SARS-CoV-2 cases in the hospital setting broadly reflected that in the community, as the great majority of infections occurred during March 2020 (87.7%). However, in April 2020, a sharp decrease in the curve was observed in the hospital, while the community continued to be severely affected in April and May [15].

During the first epidemic wave of SARS-CoV-2, hospital personnel in Brescia had an incidence of 5.9%. In a recent meta-analysis, the estimated prevalence of SARS-CoV-2 infection in HCWs was 11%, although high variability was detected among the evaluated studies (from 0.4 to 57%) [16]. The inclusion of nHCWs in our study may explain the lower incidence seen compared to others [6, 9]. Since the beginning of the epidemic, home-working strategies have been implemented to reduce the risk of transmission. This intuitively contributed to the lower incidence detected in nHCWs (*p* < 0.05). Moreover, hospital directives released on 5th March 2020 did not indicate the need of PPE for hospital personnel not directly involved in assistance to COVID patients, following the indication of the World Health Organization at that time [17, 18]. Nevertheless, the hospital offered surgical masks to both HCWs and nHCWs, and FFP2/FFP3 in case of direct assistance to COVID patients. Universally, PPE utilization was progressively introduced on 23rd March, although not mandatory but highly suggested, and became required for everybody on the 18th of May [19, 20]. Interestingly, nHCWs were much less tested compared to HCWs, but had a slightly higher positivity rate (19% vs 15.2%). This might be explained by the fact that nHCWs were a lower priority for PPE distribution (PPE not indicated unless in case of front-office activities until the 23rd March) and nHCWs might also have had a lower perception of the risk. HCWs, feared of being a possible source of infection, self-established very early quarantine measures, limiting contact with relatives and friends far beyond this became mandatory for the general population. This attitude may have reduced unprotected exposure to unknown infected people in community settings, especially in the early epidemic. The impact of in-hospital versus community-acquired infection is still to be well understood, and some authors highlighted the possibility of community transmission for HCWs too [16]. However, the importance of workplace exposure had already been suggested by other studies [16]. In our survey, hospital staff had a significantly greater incidence compared to the community (5.9% versus 1.1%). Medical doctors (8.9%) and nurses (8.5%) were at higher risk of being affected by SARS-CoV-2 compared to other professional categories. Moreover, personnel involved in COVID dedicated units had more than double the risk of being infected with SARS-CoV-2 compared to those working in non-COVID units (OR 2.74). This is in contrast with studies performed in Wuhan at the beginning of the epidemic which showed a higher risk of infection in low-risk units, where workers were less trained on the use of PPE [6, 21]. Conversely, more recent Italian [22–24] and American studies [25] showed results similar to ours, alongside the Denmark study on seroprevalence (thus identifying also asymptomatic cases) that demonstrated a higher prevalence for workers in COVID-19 units than other frontline workers [26]. Besides, another retrospective survey conducted in our hospital found no correlation between working in COVID units and positive serostatus, presumably due to a higher risk awareness [27].

At the very beginning of the epidemic, the time investigated by our study, transmission routes of SARS-CoV-2 had not been fully elucidated and risk mitigation measures (i.e., specific distinction of departments in COVID-19 units and non-COVID-19 units, proper use of PPE) had not been established yet. These factors facilitated in-hospital transmission in our Infectious Diseases Department, the first one accepting confirmed and suspected COVID-19 patients.

Conversely, the steep decrease of positive cases among hospital personnel observed after March probably reflects adequate preventive and tracing nosocomial measures implemented on daily bases, while new scientific evidence was cumulating [21, 28–31].

Finally, the perception of those who answered the questionnaire suggests that infections were mainly acquired from the working environment (88% of 279 positive interviews), where at least 40% of cases were reportedly acquired by contact with colleagues. The fact that about one-third of 213 interviewees reported not to have worn any masks at the time of presumptive contagion supports the important role of masks in preventing infection spread [28–31].

The role of asymptomatic and pre-symptomatic infections in transmission was debated at the time of the investigation. More recent evidence supports the role of asymptomatic cases in the transmission of the infection [32, 33]. In this scenario, risk mitigation measures adopted in hospital settings have certainly reduced transmission from asymptomatic or pre-symptomatic affected people. The risk of asymptomatic transmission was unknown in the early epidemic, and this could have determined a false perception of safety among employees when interacting with each other, especially in the few and short breaks during highly stressful shifts.

Our study has limitations. First, its retrospective design limited the number of variables in this study. Second, we did not detect asymptomatic infections and the real infection rate in the whole cohort might have been underestimated. Third, the results of the questionnaire analysis may have been biased by individual beliefs and perceptions on the time and site of exposure. Finally, the findings of the questionnaire may be biased by the proportion (about 50 of non-respondents).

## Conclusion

We report evidence of great risk of acquisition of SARS-CoV-2 among health-care workers at the very beginning of the epidemic. The infection spread very quickly among the clinical personnel before mitigation measures were recommended and implemented. However, the early flattening of the curve of infection of in-hospital staff, compared to the general population, suggests that preventive interventions were effective. As expected, health-care workers of COVID dedicated units presented a significantly higher risk of contracting SARS-CoV-2. Our data show that the adoption of preventive measures together with a supply of adequate PPE played a pivotal role in containing hospital transmission.

The SARS-CoV-2 epidemic of 2019 warns us about the risk for health personnel at the onset of epidemics due to new agents. Effective preparedness policies should always be rapidly implemented to protect people and places of care.
